# The Institutional Worker

**Published:** 1919-11-01

**Authors:** 


### INSTITUTIONAL WORKER

Being a Special Supplement to "The Hospital"

### OUR BUREAU OF INFORMATION.

### Rules for Correspondents.

1. Every letter must be accompanied by the coupon to be cut from the back cover (inside page) of THE HOSPITAL, current issue, and must contain the name and address of the correspondent with pseudonym for publication if desired. Replies by post cannot be given save under exceptional circumstances at the Editor's discretion.

2. Letters from Approved Homes in reply to special needs published in the Bureau should state terms and full particulars, and be sent prepaid under cover to the Editor of the Bureau with name written across coupon for identification.

3. Proprietors of Homes which have not yet been entered on the List of Approved Homes, but have spare accommodation likely to suit special needs, are invited to write for an application form for registration. The fee for registration, which includes two announcements of the Home in the Bureau and other privileges, is 10s.

4. All communications to be addressed to the Editor of THE HOSPITAL, 28 Southampton Street, Strand, London, W.C. 2, and marked "Bureau of Information."

### INSTITUTION FACTS AND FIGURES.

### Cost of Private Infectious Hospital Case.

### Answer to August Question.

By "Wales."

THE question was :

**Give the actual cost of a private patient (occupying a side ward or cubicle) in an infectious hospital, where the patient has no special nurses. State clearly the cost of food, laundry, disinfection, ambulance, nursing, and medical attendance, etc., and on what each item is based.**

The actual cost of a private patient in an infectious hospital would work out on the lowest possible basis at about £2 15s. 6d. at the present time. Roughly speaking the following expenditure would be necessary :

FOOD.

This would vary. During the first two weeks the patient would practically live on fluids, but remembering he would need feeding up during convalescence, an average allowance of 16s. 6d. per week would not be exorbitant.

| | s. | d. |
|---|---|---|
| Bread (weekly allowance, 5 lb. at 4½d. lb.) ... ... | 1 | 10½ |
| Milk (1 quart daily, including puddings, cocoa) ... | 5 | 3 |
| Butter (2 oz. weekly) ... | 0 | 3½ |
| Margarine (6 oz. weekly) ... | 0 | 4 |
| Sugar (½ lb. weekly) ... | 0 | 3½ |
| Eggs (four at 4d.) ... ... | 1 | 4 |
| Meat (four times a week) ... | 2 | 8 |
| Fish (three times a week) ... | 2 | 3 |
| Tea (2 oz. weekly) ... ... | 0 | 2½ |
| Extras .. ... ... ... | 2 | 0 |
| | 16 | 6 |

LAUNDRY.

This would cost about eighteen-pence a week, providing the washing is done on the premises.

| Average per week. | s. | d. |
|---|---|---|
| Seven sheets at 1d. ... ... | | 7 |
| Three pillowslips at 1d. ... | | 3 |
| Four towels at 1d. ... ... | | 4 |
| Four nightshirts at 1d. ... | | 4 |
| | 1 | 6 |

As the patient becomes convalescent fewer sheets and pillowslips would be wanted. The counterpane would need changing about once in three weeks.

The remaining average weekly costs would be :—

| | s. | d. |
|---|---|---|
| Ambulance, depending on distance, say ... ... | 7 | 6 |
| Disinfecting ... ... | 5 | 0 |
| Doctor and Medicine ... | 12 | 6 |
| Nursing ... ... ... | 10 | 6 |
| | £1 15 | 6 |
| Food ... ... ... ... | 16 | 6 |
| Washing ... ... ... | 1 | 6 |
| Making total weekly cost | £2 13 | 6 |

An ordinary case needs little medicine beyond gargles and aperients. This leaves a balance of 2s. for coal, etc.

### Reconstructing a Hospital.

By F. S.

THERE are doubtless many hospitals faced at the present time with the problem of modernising their buildings and plant to meet the great advances in medical and surgical science made in the past five years. A brief report upon how a Midland hospital faced the problem of reconstruction on a large scale may be of timely interest to our readers.

As the result of a report by the hospital architect on the general condition of the buildings, a Reconstruction Committee was appointed to consider the question of bringing the institution up to date by reconstruction and extension of the present premises. The Committee consisted of members of the Committee of Management and Medical Committee. Expert architectural and engineering advice was availed of, and the heads of the administrative, medical and nursing staffs consulted on matters concerning their particular departments. After a period of two years' hard work, a final report was made to the Committee of Management, who submitted it to an eminent firm of hospital architects for their comments and suggestions.

A block plan was first drawn up showing both land available and land likely to be acquired. The departments found to require consideration included the following : Two blocks, each consisting of two wards, isolation block, children's wards, out-patient department, laundry, disinfecting plant, central heating station, pathological laboratory, mortuary block, etc. These units were then placed in order of their particular urgency and their relationship to the scheme as a whole. It should here be stated that in order to meet the constant changes in hospital constructional methods, general plans only were decided upon, the details being deferred until the time arrived for actual erection. Portions of the existing building will be available for other purposes as the scheme progresses; their conversion into additional theatre units, residential quarters, etc., is arranged for.

Practical illustrations of the benefit of the scheme may be cited. A disinfecting plant was urgently wanted and had to be acquired before the site allocated to it was available. The plant was therefore temporarily housed until the permanent building could be erected. Again, a new telephone system recently installed allows for the necessary extensions to units not yet in existence. The first unit to be erected was a block of two wards, and the opportunity was then taken to inaugurate a reconstruction fund.

### Question for November.

### Reducing Hours without Increasing Staff.

**State how you would deal with the problem of reducing the weekly hours of the Male Manual Staff of a Hospital from say 60 to 48 without increasing the number of such Employees. There are 6 Porters, 2 Boilermen, 1 Laundryman, 3 Gardeners (one of whom assists inside), and a Boy. The Hospital is built upon the pavilion plan, has ong corridors (glass roofs and sides) and large grounds. All window-cleaning is done by the Porters. There are 2 steam and 4 hot-water boilers, the Heating System not being central. 187 beds.**

**(A minimum payment of 10s. 6d. will be made for each published answer.)**

RULES.

The following rules must be observed:—

1. Contributions must be written on one side of the paper. Conciseness and terseness are desirable features. The MS. must bear the name and address of the sender and be accompanied by coupon to be cut from the back cover (inside page) of the current issue of THE HOSPITAL. A pseudonym must be chosen if the name is not to be published.

2. Contributions must be addressed to the Editor of THE HOSPITAL Institutional Worker Supplement, 28 & 29 Southampton Street, Strand, London, W.C. 2, should reach him before the end of the current month, and be marked in the left-hand corner "Facts and Figures."

### QUESTIONS INVITED.

IN connection with our Question Box, we offer every month five shillings each for the best questions which are sent in for consideration. The questions may be on any institutional subject and concern any department, from the secretary's to the porter's, or the matron's to the domestic staff; the one essential is that they must be practical and deal with points that have a definite relation to hospital or institutional administration, upkeep, finance, or management. Points relating to artificers' work, housekeeping, the laundry, out-patients, and other practical matters will be welcome. It is hoped that thus, when difficult or doubtful points arise in the course of their work, institutional workers will be encouraged to put them in the form of a question and send them to the Editor, THE HOSPITAL Bureau of Information, 28 & 29 Southampton Street, Strand, London, W.C. 2, marked "Question Box."

The best questions will be published in due course and our readers will be given the opportunity of answering them. Thus all institutional workers may be able to co-operate with the view of helping the work and smoothing the difficulties of each other. In short, we want the Question Box of the *Institutional Worker* to be freely used by every worker habitually.

### ENQUIRIES AND ANSWERS.

### *EMPLOYMENT AND TRAINING.*

**Thoroughly Qualified Dispenser.**

THE highest degree for a woman dispenser is that of pharmaceutical chemist, which necessitates a long course of study—at least three years, and the whole of that time must be spent with a chemist either in a shop or the dispensary of some institution. A Preliminary Arts examination must be passed before commencing apprenticeship. The outlay involved would be approximately £100. You could qualify to work under a doctor only by passing the Assistants' Examination of the Society of Apothecaries of London, for which six to twelve months is sufficient time for study. If you decide to go in for the higher degree write for fuller particulars either to the Pharmaceutical Society, 17 Bloomsbury Square, W.C., or to Miss Buchanan, Gordon Hall, Gordon Square, W.C. If you choose the assistants' qualification, write to the Secretary, Pharmaceutical Society, Bloomsbury Square.—A. B.

**Infant Welfare Work.**

WE suggest that you write to the Secretary of the National Association for the Prevention of Infant Mortality, 4 Tavistock Square, London, W.C., who will probably advise you as to your best method of obtaining the work you desire. You might also write to the Matron of the Southwark Municipal Nursery, Southwark, S.E. Enclose a stamped addressed envelope for reply in each case.—E. K. P.

**Address Required.**

THE address of the Overseas Nursing Association is Imperial Institute, London, S.W. Write also to the British Women's Emigration Association, The Imperial Institute. We decidedly advise you not to go out without first obtaining definite work to go to. It will depend upon the arrangements made between you and the authorities as to whether you pay your own passage or not.—E. M. M.

### *THE SICK AND IN NEED.*

**Convalescent Home for Women.**

LONDON cases are received at the Rhyl Women's Convalescent Home, Morfa Hall, Rhyl. Admission may be by nomination and payment or by payment only. A weekly payment of 15s. is required if the patient does not obtain a letter of recommendation—this charge may be slightly increased now. Write to the Secretary for a form of application, who will also give you full details. Failing this you might apply to the Sister Superior, St. Raphael's Convalescent Home, Higher Lincombe Road, Torquay. London cases are also received here upon payment of 12s. a week in advance; washing extra.—S. B.

**Epileptic Colony in London.**

WE suggest the County Epileptic Colony, Ewell, near Epsom, Surrey, for the epileptic man. Patients must, however, be sufficiently able-bodied for employment. Write, giving full particulars of the case, to the Medical Superintendent, who will, no doubt, advise you.—F. H.

**Invalid Child.**

CHILDREN between three and eight years are taken in the Heritage Preparatory School, Guild of Brave Poor Things, for treatment and teaching. An annual payment of £36 is required. It is possible that the child suffering from heart disease might be admissible. Apply to the Hon. Secretary, Guild of Brave Poor Things, The Chapter House, Southwark Cathedral, S.E.—MISS G.

### *MISCELLANEOUS.*

**Prospects of Nurses.**

THE College of Nursing has, as a result of expressions of dissatisfaction existing in many sections of the nursing profession, made exhaustive enquiries as to the salaries and conditions of employment of nurses in institutions and other spheres of work throughout the country. A special Committee was appointed by the College for this purpose, and they have recently issued a report in which much useful and interesting material is carefully tabulated. We recommend you to obtain a copy of this report from the College of Nursing, Ltd., 7 Henrietta Street, Cavendish Square, W. 1. The price is 6d.—MATRON.

**Addresses Wanted.**

THE addresses you ask for are: National Children Adoption Association, 11 Sloane Street, S.W. 1; National Council for the Unmarried Mother and her Child, Evelyn House, Oxford Street, W. 1.—T. A.

**Rat Plague.**

THE destruction of rats is a question upon which the Board of Agriculture could advise you. The Board is promoting a campaign against the pest, and have organised the National Rat Week. The subject, allied with other household pests, is one which we have referred to on many occasions in these columns. The remedy of constructional defects is perhaps the surest way of getting rid of the trouble, though just now this might involve you in a greater expense than you can afford. If you apply to the "Rats" Branch Department of the Board of Agriculture, Whitehall Place, S.W. 1, you will be informed as to where you can obtain the approved kind of rat-destroying materials.—W. G. T.

